# Retraction Note: Therapeutic effect of molecular hydrogen in corneal UVB-induced oxidative stress and corneal photodamage

**DOI:** 10.1038/s41598-021-89085-8

**Published:** 2021-04-23

**Authors:** Cestmir Cejka, Jan Kossl, Barbora Hermankova, Vladimir Holan, Sarka Kubinova, John H. Zhang, Jitka Cejkova

**Affiliations:** 1 Institute of Experimental Medicine of the Czech Academy of Sciences, 14220 Prague 4, Czech Republic; 2 Faculty of Natural Science, Charles University, Vinicna 7, 12843 Prague 2, Czech Republic; 3 Loma Linda University School of Medicine, Loma Linda, CA 92350 USA

Retraction of: *Scientific Reports* 10.1038/s41598-017-18334-6, published online 21 December 2017

The Editors have retracted this Article.

An institutional investigation found evidence that the H2 condition for Figure 3B has been duplicated from Figure 5C of an article previously published by Cejka et al. (2016)^1^ and the day 4 H2 condition for Figure 4A has been duplicated from Figure 3 of an article published later by Cejka et al. (2020)^2^. The authors have not been able to provide all the relevant raw data on request.

The Editors therefore no longer have confidence in the accuracy of the reported data and the conclusions of the Article.

Sarka Kubinova agrees with the retraction and its wording. Cestmir Cejka, Jan Kossl, Barbora Hermankova, Vladimir Holan, John H. Zhang, and Jitka Cejkova disagree with the retraction.
